# Observations on Pulmonary Consumption

**Published:** 1830-01-01

**Authors:** 


### Observations on Pulmonary Consumption. By Dr. Parrish.

Our readers are well aware that we have often expressed our doubts respecting the usual modes of treatment employed by medical practitioners in the scourge of the world, (for England is not the only country which it loves to ravage) phthisis pulmonalis. The paper, from which we mean to make some extracts, is from the pen of one of the most distinguished physicians of America, Dr. Parrish, and his observations deserve particular attention.

Dr. P. confines his remarks to that form of pulmonary consumption which is characterized by the presence of tubercles—and which he justly considers as essentially scrofulous in its nature, and too often dependent on hereditary predisposition. His remarks are directed entirely to the treatment of the complaint, and that based on a very long and ample experience. When first he entered the profession he was enamoured with the doctrines of Rush, who viewed pulmonary consumption as an inflammatory disease, and, consequently, employed various antiphlogistic measures for its relief, as frequent bleedings, a rigid system of diet, confinement to rooms of regulated temperature, and depressive medicines. Dr. Rush conjoined mercury. "But whatever plan was adopted, the poor patients generally went one course, and that rapidly—they died." Dr. Parrish, in the course of a few years, began to doubt the doctrines of Rush.

"My doubts were, indeed, very early excited by the opposite results of two apparently very similar cases of the disease, which occurred at the same time and in the same neighbourhood, and one of which I myself attended in consultation with Dr. Rush and another highly distinguished physician. I will relate these cases, as strongly illustrative of the inefficacy and injury of vigorous medical treatment in this complaint.

"The patient whom I attended was a young man, not more than twenty-four years of age, the eldest child of an anxious and affectionate father, whose wealth enabled him to command every possible assistance, and who, having lost a wife with pulmonary consumption, was alarmed by the first appearance of the symptoms in his son, so that no delay was incurred in commencing the treatment, and no means were spared which it was thought might be productive of advantage. As it was in the beginning of winter that we were called to the patient, our first object was to obviate the effects of the weather: we therefore had him placed in a spacious apartment, the air of which was maintained at a uniform temperature by night and day throughout the season. This was effected by means of a soapstone stove, and a thermometer suspended in the room. The treatment at first consisted in a system of rigid dieting, with small and frequent bleedings and the use of mercury. Ptyalism, however, could not be induced; and as the patient grew worse under the present plan, we laid aside the mercury, and resorted to diaphoretics. Sulphur and tar-water were also prescribed, under the impression that they had been useful in similar cases. But our remedies appeared to make no impression on the disease, which marched steadily forward. Various medicines were afterwards used with little advantage; among the rest, acetate of lead, which was given in the dose of two grains every two hours for several days in succession, with the effect of diminishing the frequency of the pulse; but as it induced symptoms of colic, we were under the necessity of abandoning it. At this period I visited the patient twice every day, and my two colleagues every morning; and nothing was omitted which occurred to the experience or sagacity of those highly distinguished physicians as likely to be productive of benefit. At length that stage of the disease arrived in which a supporting treatment was thought to be required. A stimulating diet, with tonics of various kinds, was now resorted to; but all our efforts were unavailing. The patient came under our care in the early part of winter and died before the close of the following spring.

"In the immediate neighbourhood of this young gentleman resided a student of medicine, afterwards a respectable practitioner. In the preceding summer, while resident pupil in the Pennsylvania Hospital, he had been attacked with hæmoptysis, in the treatment of which a vigorous course of depletion had been pursued. The hæmoptysis disappeared, but was followed by considerable debility. A visit to the country did not restore his health, and he returned in the fall too unwell to resume his duties in the hospital. At the commencement of winter, any one, upon observing the two patients, would have supposed that the student was further advanced in the disease, more reduced, and more likely to pass away than his unfortunate neighbour. He was pallid, emaciated, had cough and fever, in fact, exhibited all the marks of confirmed consumption. He resisted, however, all attempts to induce him to submit to medical advice, from a belief that the practice which would be adopted, would tend only to hasten a fatal issue. The winter passed with no other treatment than the occasional use of slight palliatives, as paregoric to allay cough; and the spring which saw our patient carried to the grave, opened upon this young gentleman still alive. As soon as the weather permitted, he went into the country, and furnishing himself with a horse, sick and debilitated as he was, commenced the life of a country doctor. Strange as it may appear, he rode himself into perfect health. He acquired an extensive practice, married, and became the father of several children. He afterwards returned to the city, and fell a victim to typhus fever, contracted during his attendance upon the business of the dispensary. This happened ten or twelve years after the winter above alluded to; and not a symptom of his former complaint was observable for a long time before the period of his death. I often conversed with this gentleman relative to his case, and remember being told by him, that he found no remedy so effectual in relieving his distressing chilliness as a ride on horseback. In the midst of a chill, while sitting by a large fire, having the back of his chair covered by a thick coat or blanket, and yet unable to keep himself warm, he would receive a message from a patient at a distance requiring him to mount his horse. Almost immediate relief would be experienced from the exercise, and a ride of a few miles would produce so much excitement as to restore him to comfortable warmth."*

The doctor had frequent opportunities afterwards of observing the injurious effects of rigid antiphlogistic treatment —and its almost invariably fatal result. He at length came to the conclusion, that neither depletion, nor any medicine, nor any combination of medicines, would cure the disease, or even materially alleviate the symptoms.

"I recollect a case of pulmonary consumption, in which a rigid antiphlogistic diet, adhered to for some time, had of itself the effect of producing a great increase in the excitement of the pulse, which was allayed by a change to more nourishing food. The circulation even of a healthy individual may be brought into an irritated state by depriving the system of that support from a due degree of food, air, and exercise, which is essential to the preservation of a just balance in all its operations. Take a robust man, confine him in a close room, bleed him repeatedly, diet him strictly, keep up action in his bowels by purgative medicine, and allow not a breath of air to blow upon him, and, if I am not greatly mistaken, his pulse will become frequent and irritated, with the occurrence of night sweats, and perhaps sizy blood; in fact that very condition of system will be produced, for which, in cases of phthisis, these measures are recommended as remedies. Now it is well known, that in consumptive patients there is generally a preternatural irritability; and it is reasonable to infer that this irritability must be augmented

* North American Medical and Surgical Journal, No. XVI.

by the means which are sufficient to produce it in a healthy man; so that the system will thus be rendered less capable of resisting the operation of morbid causes, and may sink under the local disease which it might have otherwise withstood for many years, perhaps ultimately have surmounted.

"But, though the antiphlogistic course of treatment, and the use of powerful medicines, are calculated rather to co-operate with the disease of the lungs in reducing the system of the consumptive patient, than to relieve or eradicate the complaint, yet we are not therefore to surrender all hope, and yield up the sufferer an unresisting victim. On the contrary, much may be done by exertion on the part of the individual affected in controlling the disease; and instances are not wanting in which it would seem to have been entirely conquered.

"Vigorous exercise, and free exposure to the air, are by far the most efficient remedies in pulmonary consumption. It is not, however, that kind of exercise usually prescribed for invalids —an occasional walk or ride in pleasant weather, with strict confinement in the intervals,—from which much good is to be expected. Daily and long continued riding on horseback, or in carriages over rough roads, is, perhaps, the best mode of exercise; but where this cannot be commanded, unremitting exertion of almost any kind in the open air, amounting even to labour, will be found highly beneficial. Nor should the weather be scrupulously studied. Though I would not advise a consumptive patient to expose himself recklessly to the severest inclemencies of the weather, I would nevertheless warn him against allowing the dread of taking cold to confine him on every occasion when the temperature may be low or the skies overcast.

"I may be told that the patient is often too feeble to be able to bear exertion; but, except in the last stage, where every remedy must prove unavailing, I believe there are few who cannot use exercise without doors; and it sometimes happens, that they who are exceedingly debilitated find, upon making the trial, that their strength is increased by the effort, and that the more they exert themselves, the better able they are to support the exertion.

"It is said by those who oppose this kind of treatment, that the lungs are in a state of inflammation, and therefore require rest. But, admitting for a moment that the tubercles in pulmonary consumption are the result of ordinary inflammatory action of a chronic or subacute character,—an opinion, however, which I have already disclaimed, —yet the argument will not hold in the present case; for, from their very organization, the lungs cannot be at rest. From the moment we begin to breathe to the latest period of life, they are necessarily in continual motion. We cannot confine them as we can a diseased joint; and in attempting to restrain their motions by keeping the body at rest, without gaining our object, we generate a degree of irritability of system, which enables the local affection to operate upon the general health with a vast increase of deleterious effect.

"I was much pleased to meet with a confirmation of my views relative to the management of pulmonary consumption, in the following extract from a work of Dr. Colin Chisholm, whose experience entitles his opinion to great respect. 'An active, bustling occupation of time,' he says, 'with exposure to what may be called and deemed hardships; such as occur in military service during an active campaign, or in maritime service of any kind; have sometimes produced a most wonderful change in a constitution broken down by phthisis. I have known instances of officers in both services recovering their health by seemingly inconsistent means. *One thing is most certain, that confinement to the atmosphere of a room, or even house, is most highly prejudicial:* it renders the person infinitely more susceptible of the impressions of cold, and thereby tends to augment the evil which it is supposed calculated to remedy."

Dr. Parrish next relates a few instances where this mode of life was attended with beneficial consequences. We shall rapidly glance at some of these.

"The late Dr. Baldwin had a strong hereditary disposition to pulmonary consumption, having lost a father and several brothers by the disease, and being himself attacked with it soon after he graduated, and while a resident at Wilmington. Aware of the inefficacy of the usual treatment, he determined to try change of climate and great bodily exercise. In the midst of winter he embarked for Savannah, and on his arrival there, he set off on foot for MILLEDGEVILLE, the capital of the state, one hundred miles distant. His friends at Savannah considered the attempt as madness, his health was in such a dreadful state.

"Diregarding, however, their representations, he took from his trunk some necessary articles of clothing, tied them in a handkerchief, ran a stick through the bundle, and placing it on his shoulder, set off on his pedestrian journey. As the country was but thinly settled, he endured many hardships and privations, being sometimes compelled to wade through streams, and often taking up his lodging in cabins, among people as untutored as the Indian, and partaking of their homely fare of ham and corn bread. Arriving safely at the capital, he there met with Colonel Hawkins, agent for the United States among the southern Indians, by whom he was treated with great attention, and invited to accompany him to his residence. He accepted the invitation, and having passed the winter at the Agency, found himself, upon the opening of summer, nearly restored to health."

The disease was suspended for six years, when it again assailed him, and he sunk a victim to the insatiate enemy. The following extract, though not exhibiting what might be termed specific and unequivocal evidence, is yet well worthy of perusal and consideration.

"A young physician, who had been a pupil of my own, was, soon after entering into practice, attacked with fistula in ano, for which I operated upon him. The fistula was small, but much indisposed to heal; and a long time elapsed before a cure was effected. Very soon afterwards he was attacked with hæmoptysis, from which he had scarcely recovered, when the symptoms of consumption became manifest. I watched the progress of the case with great solicitude. Necessity compelled him to use great exertion to gain a livelihood, and I was often surprised to find him confined by an attack of spitting of blood, and a few days afterwards running about among his patients in the lanes and alleys of the city. I am convinced that his life was protracted at least one year longer than it otherwise would have been, by the great exertion to which the stimulus of necessity excited him.

"Similar effects I have repeatedly witnessed in other cases. During my attendance in the Philadelphia Dispensary, my attention was occasionally attracted by patients affected with consumption, whose situation in life left them only the alternative of labouring for their support, or of becoming tenants of the Alms-House. The strong aversion, entertained by many of the poor to entering this institution, serves as a powerful incentive to exertion; and I remarked that some patients, who were by this cause induced to struggle by their labour for a support to the very last, continued longer, and bore their disease better than others, whose circumstances were considered much more comfortable.

"From a fine, healthy-looking practitioner of New England, who some years ago brought me a letter of introduction, I received the following account. In early life he had been affected with pulmonary consumption, and, while still labouring under the disease, had commenced the life of a country doctor. As there happened to be an uncommon degree of sickness in the neighbourhood at this period, he was compelled to make great and unusual exertion; and the result was a perfect restoration to health. In the course of conversation he said to me (I copy his words); 'I have left a patient at home labouring under pulmonary consumption, with directions to ride ten miles every day, be the weather what it may.' This was in the winter season.

"The following notice, handed me by Dr. GILMAN, was drawn up by a gentleman of Ohio, the father of the Doctor, and dated Marietta, 1823. 'In the year 1804, Thaddeus M. Harris, a clergyman of Massachusetts, called at my house in Marietta, and from him I received the following account. He had left Dorchester, Massachusetts, that spring, so low in consumption, that neither he, nor any of his friends had an idea that he would be able to reach Hartford, Connecticut, distant one hundred miles. He arrived there, however; and, though still very weak, he was encouraged to prosecute his journey to New York. When there, finding that he was gaining strength, he concluded to proceed to the western country. On his arrival at Marietta, he was so well as to be able to ride forty miles a day to preach, and was in fact quite recovered. He returned to his parish in Dorchester in good health; and the last time I heard from him, which was about two years since, he was still well.'

"A gentleman of this city, when a young man, came under my care, during the winter season, affected with cough and hectic fever. I felt great solicitude in his case, and determined to try the effect of horseback exercise. In compliance with my advice, he rode daily through the winter, and in the spring was evidently improved. The summer opened upon him still affected with alarming symptoms. It was during the late war; and in the course of the season camp Dupont was formed. The young man joined one of the volunteer companies, marched down with the rest, and was subjected to all the hardships of a camp life. His health and strength increased; and he is now a hearty man, free from all signs of pulmonary disorder.

"Another very interesting case occurred to me with a similar result. A little son of a respectable citizen of Philadelphia was affected with cough, hectic fever, profuse sweats, and great emaciation; and there was every reason to believe that his lungs were affected with tuberculous disease. Entertaining the conviction that any active medical treatment would insure a fatal termination to the case, I strenuously opposed the use of remedies which were pressed upon the parents by the kindness of their friends. Among the rest mercury was proposed. Happily they were disposed to listen to the suggestions of their physician, and no active treatment was employed during the winter. At the opening of spring, the child was sent into the country, with directions that he should have the benefit of free exercise, and the open air. He returned free from the pulmonary complaint, has since passed through the hooping-cough and remains well to this time.

"I shall close this list of cases, by giving one more illustration of the comparative effects of exercise, and of confinement, in the cure of consumption. Shortly before the death of Dr. WISTAR, an interesting young lady was brought from New Jersey, to consult him for chill, fever, pain in the breast, cough, and considerable loss of voice. These symptoms presented a gloomy prospect. The Doctor being absent on a journey when the lady arrived, she came under my care; and I had seen her several times before his return. We afterwards visited her together, and made a very minute examination of her case. Dr. WISTAR having been travelling over the mountains, in which mode of life he took great delight, was fully prepared to appreciate the effects of air and exercise. We had retired for the purpose of a consultation, and had re-entered the apartment, when looking round the room, he made this remark to me: 'Doctor, do you not think it looks confined and close here? Do you not think it would be best to send her back to the country, and direct her to ride every day?' I concurred heartily in the proposition. We put a seton in her side, and then advised her to return home, and use exercise. The winter passed over. The following summer she visited Philadelphia greatly improved in health, and, as I afterwards learned, became perfectly well."

Dr. Parrish concludes by expressing his conviction that, "in the management of consumption, no remedies are so efficient as fresh air and active continued exercise." Speaking of the practice usually pursued in this disease, he gives it as his decided opinion that, "if patients affected with phthisis were universally left to themselves freely in the open air, the general result would be a longer continuance of life, and a greater number of recoveries."

Without concurring, to the full extent, in the opinions of Dr. Parrish, and without having so much confidence in air and exercise as he seems to have, we believe, from some curious facts within our knowledge, that there is considerable foundation for what he advances. If, in health, we find regular exposure to the open air the very best *preservative* against coughs and colds, there is reason, from that very fact, to think that a careful exposure to the same, gradually commenced and steadily persevered in, might, in some cases at least, exert a *curative* influence. It is abundantly evident that this experiment requires great caution, and a sound discretion. The system of "air and exercise" should be commenced in the summer, and in the most gradual manner, imperceptibly augmenting the amount of each. By the approach of Winter it will be ascertained whether the plan is likely to agree, and if it does, the system may be pursued, to a greater or less extent, through the Winter and Spring. The writer of this article has had many opportunities of ascertaining and experiencing the astonishing influence of exposure to the open air, as a preservative against colds, and many other complaints. Between the 5th September and the 5th December of the past year, he travelled 3500 miles in the open air, by night and by day—in hot, cold, dry, rainy, and even snowy weather, without once catching cold, or any other complaint. He has experienced the suffocating sirocco in the forenoon, the chilling tramontane in the afternoon—the heavy dews of the evening—and a storm of sleet and snow before the morning—all in the course of 24 hours—and all in the open air, without the slightest bad effect; but, on the contrary, with a kind of certitude or conscious feeling of being completely proof against aerial vicissitudes. An example or two may not be quite uninteresting.

On the 2d November, at Naples, the sirocco had blown all the preceding night, and till nine o'clock in the morning, with a most suffocating warmth and humidity that rendered life a misery. At ten o'clock the wind chopped round to the north-east, and the writer set off in an open carriage for Herculaneum and Pompeii. The breeze was very strong, and, in the whole course of his life, he scarcely ever felt the cold more intense than during the drive to Pompeii. While wandering among the ruins of that disinterred city, he was alternately scorched with a still powerful sun, and chilled with the tramontane blast, according as locality placed him in a northern or southern aspect. To this was added fatigue as well as hunger. The whole day was spent among the ruins, and the journey back to Naples was in the night, with heavy dews descending; yet no cold was caught, nor ill consequence suffered. A more severe trial was undergone a fortnight afterwards. On the 16th November he slept at a small town called FINALE, between Genoa and Nice, on the Mediterranean shore. He was travelling in a little open calesh, with one horse and a guide, at this time. The next day's journey to St. Remo was a long and rugged one, and it was necessary to start at four o'clock in the morning. During the night, the formidable BIZE wind set in with great force, and the rattling of the doors and window-shutters of FINALE, precluded all attempt at sleep. At half-past four o'clock in the morning of the 17th November, they were winding their way, in the dark, up a zig-zag path in the mountain, along a precipice of a thousand feet perpendicular above the sea, and with overhanging rocks, of one or two thousand feet above them. The gale gradually increased as they ascended, and before they had reached the highest point, it was a perfect hurricane, and the road completely obscured by snow. The guide's courage failed, and he proposed to turn back, as the road was devoid of parapet, and not above 12 or 14 feet in breadth. The horse, however, was totally incapable of facing the storm, which was adverse to their return. They were therefore obliged to scramble onwards, at the imminent risk of being hurled over the precipice along which they were winding in the dark. The cold, the horror,—let it be confessed, the *terror*, of that morning, can never be effaced from the memory! The howlings of the wind, the roaring of the surges against the rocks beneath, the drifting of the snow, the piercing cold of the blast coming down from the Alps in fitful tornadoes, the occasional crash of fragments of rock from the impending steeps, the total obliteration of all trace of the road by the sleet—and last, not least, the thoughts of "friends and native home," while exposed to imminent peril on this frightful pass, may be imagined; but Heaven forbid that they may ever again be experienced by the benighted and way-worn traveller! By the time that day-light appeared, the poor guide was nearly dead with fatigue (being obliged to lead the horse the whole way) and the narrator was as nearly dead with cold and anxiety. When objects could be discerned, the Mediterranean beneath them was one continuous sheet of foam from the violence of the hurricane, and the whole of the surrounding mountains were covered with snow. The weary travellers had 17 miles farther to proceed before they breakfasted. With the rising sun confidence was inspired—the dread of danger vanished—the circulation returned to the half-frozen extremities—and not the slightest physical inconvenience followed the severest trial to which the narrator was exposed during a long life of wanderings.

---

